# Supplementary material for: Conduction pathway for potassium through the E. coli pump KdpFABC
Source: bioRxiv. 2025 Oct 6:2025.05.05.652293. Originally published 2025 May 6. Preprint. [Version 3] doi: 10.1101/2025.05.05.652293 (PMC12247778; doi:10.1101/2025.05.05.652293)
Supplement: 1 [file NIHPP2025.05.05.652293V3-supplement-1.pdf]

bioRxiv preprint doi: https://doi.org/10.1101/2025.05.05.652293; this version posted October 6, 2025. The copyright holder for this preprint (which was not certified by peer review) is the author/funder, who has granted bioRxiv a license to display the preprint in perpetuity. It is made available under aCC-BY 4.0 International license.

## SUPPLEMENTARY FIGURE LEGENDS

### Figure 1 - figure supplement 1. Biochemical preparation of KdpFABC.

(A) SDS gel of nanodisc sample used for cryo-EM showing the presence of KdpA, KdpB, KdpC and the membrane scaffolding protein, spNW25. KdpF is not visible due to its small size (3 kD); typically, it stains poorly and runs very near the dye front of the gel. (B) Elution profile from FPLC size-exclusion column of the sample used for making cryo-EM grids; the main peak is composed of nanodiscs containing KdpFABC and the lower peak contains empty nanodiscs. Slight asymmetry of the main peak is consistent with a subpopulation of nanodiscs containing two KdpFABC complexes (Fig. 1 - figure supplement 2). (C-D) MALS analysis of the HPLC-SEC elution profile from nanodiscs reconstituted with POPC/DOPA lipids and E. coli polar lipids, respectively. This analysis indicates DOPC/DOPA lipids produce smaller nanodiscs containing ~1 KdpFABC molecule (panel c), whereas E. coli polar lipids produce larger, more heterogeneous nanodiscs (panel d). (E) ATPase activity is preserved after nanodisc reconstitution, though at a reduced rate attributable to the lipid environment. (F) Transport current measured by solid supported membrane electrophysiology showing $K^+$ dependence of WT KdpFABC. (G) $Mg^{2+}$ dependence of ATPase activity indicates a Hill coefficient (n) of ~1.8, which is consistent with the two $Mg^{2+}$ ions associated with ADP in the structure. (H) Inductively coupled plasma mass spectrometry analysis of stock solutions used for ATPase assay indicate substantial $K^+$ contamination from ATP and $MgCl_2$ solutions. These data represent the mean and std. dev. of triplicate measurements.

### Figure 1 - figure supplement 2. Cryo-EM processing pipeline.

Four grids from a single sample were imaged to produce a total of ~50,000 micrographs. These were divided into ten groups of 2000-6000 micrographs for initial processing. After motion correction and determination of defocus parameters, particles were picked using TOPAZ and extracted with a box size of 335Å and 3-fold binning. Multiple rounds of 2D classification (a few examples of 2D classes are shown) and ab initio reconstruction were used to remove false positive picks and empty nanodiscs. A subset of these particles were further classified to generate four main classes representing nanodiscs with a single copy of KdpFABC in either E1 or E2 conformations, nanodiscs with two copies of KdpFABC (double) which were mainly E1 conformation, and junk. These low-resolution structures were used as references for hetero-refinement of particles partitioned in seven groups. Particles conforming to each conformation were combined and used for masked 3D classification, where the mask encompassed the cytoplasmic domains of KdpB. Particles representing the E1~P·ADP conformation, which represented the largest class, were combined for a final non-uniform refinement job.

### Figure 1 - figure supplement 3. Cryo-EM densities.

Correlation between map densities and model for individual transmembrane helices from KdpA as well as transmembrane helices and cytoplasmic domains of KdpB. The sharpened map was used for helices and the P-domain with a threshold of 5.7 $\sigma$, whereas the unsharpened map was used for the more flexible A- and N-domains at 2.5 $\sigma$.

bioRxiv preprint doi: https://doi.org/10.1101/2025.05.05.652293; this version posted October 6, 2025. The copyright holder for this preprint (which was not certified by peer review) is the author/funder, who has granted bioRxiv a license to display the preprint in perpetuity. It is made available under aCC-BY 4.0 International license.

## Figure 2 - figure supplement 1. Selectivity filter of KdpA.

(A) Distribution of raw densities for the entire sharpened map. The arrow indicates the threshold used for rendering the map in Fig. 2 (0.5 which corresponds to 6.5 $\sigma$). The inset shows the extreme tail in the density distribution; the highest four densities (circled at >50 $\sigma$) correspond to the $K^+$ ion modeled at the S3 site. (B) Coordination of densities in the selectivity filter in the refined model. The regular network of bonds at the S3 site suggest that this is the high affinity binding site for $K^+$, whereas less regular coordination at other sites is consistent with water.

## Figure 2 - figure supplement 2. Current traces for WT KdpFABC.

Raw data from replicate SSME titrations using WT KdpFABC are shown. The ions used for each titration are shown along the left margin. Each panel represents a single titration with ion concentrations indicated in the legend. Four replicate titrations from two different sensors were recorded for each ion. Sharp spikes in some of the traces are generally due to air bubbles in the microfluidic system and were not included in the analysis.

## Figure 2 - figure supplement 3. Current traces for Q116R.

Raw data from replicate SSME titrations using the Q116R mutant in KdpA are shown. The ions used for each titration are shown along the left margin. Each panel represents a single titration with ion concentrations indicated in the legend. Four replicate titrations from two different sensors were recorded for each ion. Transient peaks seen for $Rb^+$ and $NH_4^+$ are likely due to pre-steady state binding events and not to sustained transport of these ions. As a result, data for analysis were taken at 1.25 s, which is after this pre-steady state signal has decayed. Sharp spikes in some of the traces are generally due to air bubbles in the microfluidic system and were not included in the analysis.

## Figure 2 - figure supplement 4. Current traces for G232D.

Raw data from replicate SSME titrations using the G232D mutant in KdpA are shown. The ions used for each titration are shown along the left margin. Each panel represents a single titration with ion concentrations indicated in the legend. Four replicate titrations from two different sensors were recorded for each ion. Sharp spikes in some of the traces are generally due to air bubbles in the microfluidic system and were not included in the analysis.

## Figure 4 - figure supplement 1. Transport activity of Val496 mutants.

(A) Comparison of ATPase and transport activities from reconstituted proteoliposomes of WT and Val496 mutants. A small amount of detergent (1 mg/ml DM) was added to proteoliposomes to prevent buildup of electrochemical gradients during the ATPase assays, whereas transport was measured from intact proteoliposomes by SSME. Effects of the mutations are comparable to those seen prior to reconstitution (Fig. 4) and indicate that energy coupling is not affected. (B-G) Raw data from SSME recordings (top) as well as gel filtration profiles (bottom) are shown for WT and Val496 mutants. SSME traces represent four replicates recorded in 100 mM $K^+$ taken from two individual sensors. Note that the V496R mutant displays a highly transient peak consistent with a pre-steady state binding event, with very little sustained transport current. The gel filtration profiles characterize the final step of purification with all mutants producing a relatively symmetric peak at a consistent elution volume, thus indicating that Val496 mutation does not affect stability or homogeneity of the preparation.

bioRxiv preprint doi: https://doi.org/10.1101/2025.05.05.652293; this version posted October 6, 2025. The copyright holder for this preprint (which was not certified by peer review) is the author/funder, who has granted bioRxiv a license to display the preprint in perpetuity. It is made available under aCC-BY 4.0 International license.

### Figure 5 - figure supplement 1. Canonical binding sites of P-type ATPases.

The primary $K^+$ binding site in KdpB is structurally conserved with ion binding sites for $Na^+$ in Na,K-ATPase and and $Ca^{2+}$ in SERCA. Whereas multiple ions are transported in each cycle by these related P-type ATPases, only a single ion appears to bind to KdpB at a position equivalent to the Na2 and Ca2 sites. Like those sites, the $K^+$ in KdpB is coordinated by carbonyl oxygens exposed by the unwinding of the M4 helix near a conserved proline (Fig. 5 - figure suppl. 2). Additional ions bind in a pocket between M4, M5 and M6 in Na,K-ATPase and SERCA, which is occupied by weaker densities in KdpB that are modeled as water.

### Figure 5 – figure supplement 2. Properties of the canonical binding site in KdpB.

(A) Coordination network involving $K^+$ and water molecules in the canonical binding site. (B) Distribution of raw densities for KdpB portion of the sharpened map, with the inset highlighting the highest two densities (>30 $\sigma$), both of which correspond to the $K^+$ ion modeled at the canonical binding site (CBS). The arrow indicates the density threshold used for rendering the map in Fig. 5 (0.45 which corresponds to 5.8 $\sigma$). (C) ATPase activity of the KdpB-D583A mutant showing uncoupled activity at low $K^+$ that is inhibited at higher $K^+$ concentrations.

### Figure 5 - figure supplement 3. Current traces from CBS mutants.

Raw data from replicate SSME titrations in 100 mM $K^+$ using mutants in the CBS of KdpB. WT protein is included as a positive control. Each panel shows four replicates taken from two individual sensors and indicate very little transport activity from these mutants.

### Figure 6 - figure supplement 1. Exit site for $K^+$ from KdpB.

(A) Comparison of the canonical binding site from E1~P (this study) and E2·Pi determined previously by Sweet et al. (Sweet et al., 2021) (PDB code 7BGY). In particular, movements of M5 cause Lys586 to swing into the canonical binding site in E2·Pi, thus displacing the $K^+$. (B) Comparison of the exit site from wild-type E1~P model with a predicted model for the T75K mutant. In this predicted structure, the lysine side chain occupies the canonical binding site, thus potentially acting as a built in ion to stimulate ATPase activity in the absence of $K^+$. (C) pH dependence of both ATPase activity and transport from the KdpB-L72D mutant. Like the T75D mutant, the pH dependence of activity is shifted towards low pH. Although the rates are lower than WT and T75D, the $K^+$ dependence of ATPase activity indicates that energy coupling is intact.

### Figure 6 - figure supplement 2. Current traces from exit site mutants.

Raw data from replicate SSME pH titrations using mutants in the exit site of KdpB. WT protein is included as a control. Each panel shows four replicates taken from two individual sensors at the pH indicated across the top border. Sharp spikes in the traces are generally due to air bubbles in the microfluidic system. These spikes appear amplified for T75K due to the compressed current range of this largely inactive mutant; such spikes were not included in the analysis.

bioRxiv preprint doi: https://doi.org/10.1101/2025.05.05.652293; this version posted October 6, 2025. The copyright holder for this preprint (which was not certified by peer review) is the author/funder, who has granted bioRxiv a license to display the preprint in perpetuity. It is made available under aCC-BY 4.0 International license.

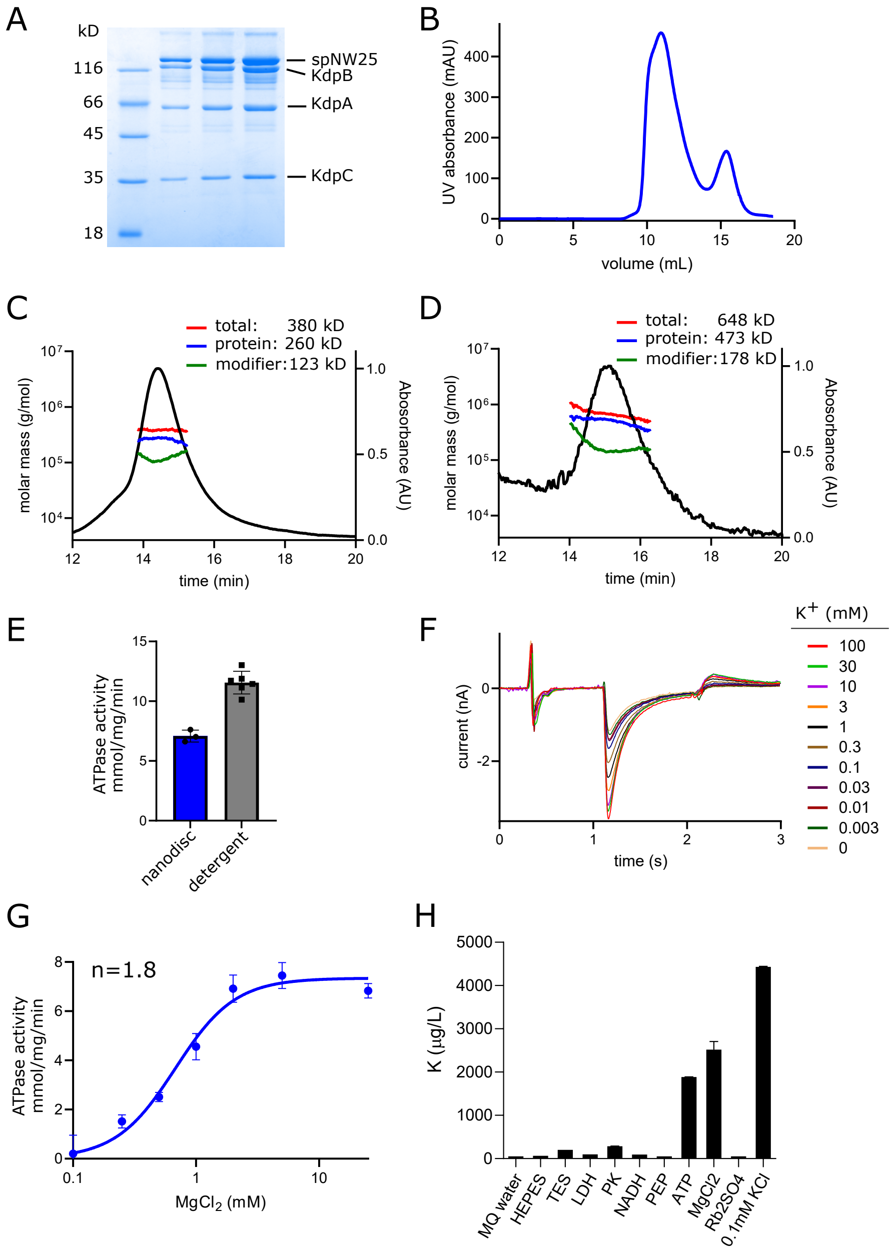


Figure 1 - figure supplement 1

bioRxiv preprint doi: https://doi.org/10.1101/2025.05.05.652293; this version posted October 6, 2025. The copyright holder for this preprint (which was not certified by peer review) is the author/funder, who has granted bioRxiv a license to display the preprint in perpetuity. It is made available under aCC-BY 4.0 International license.

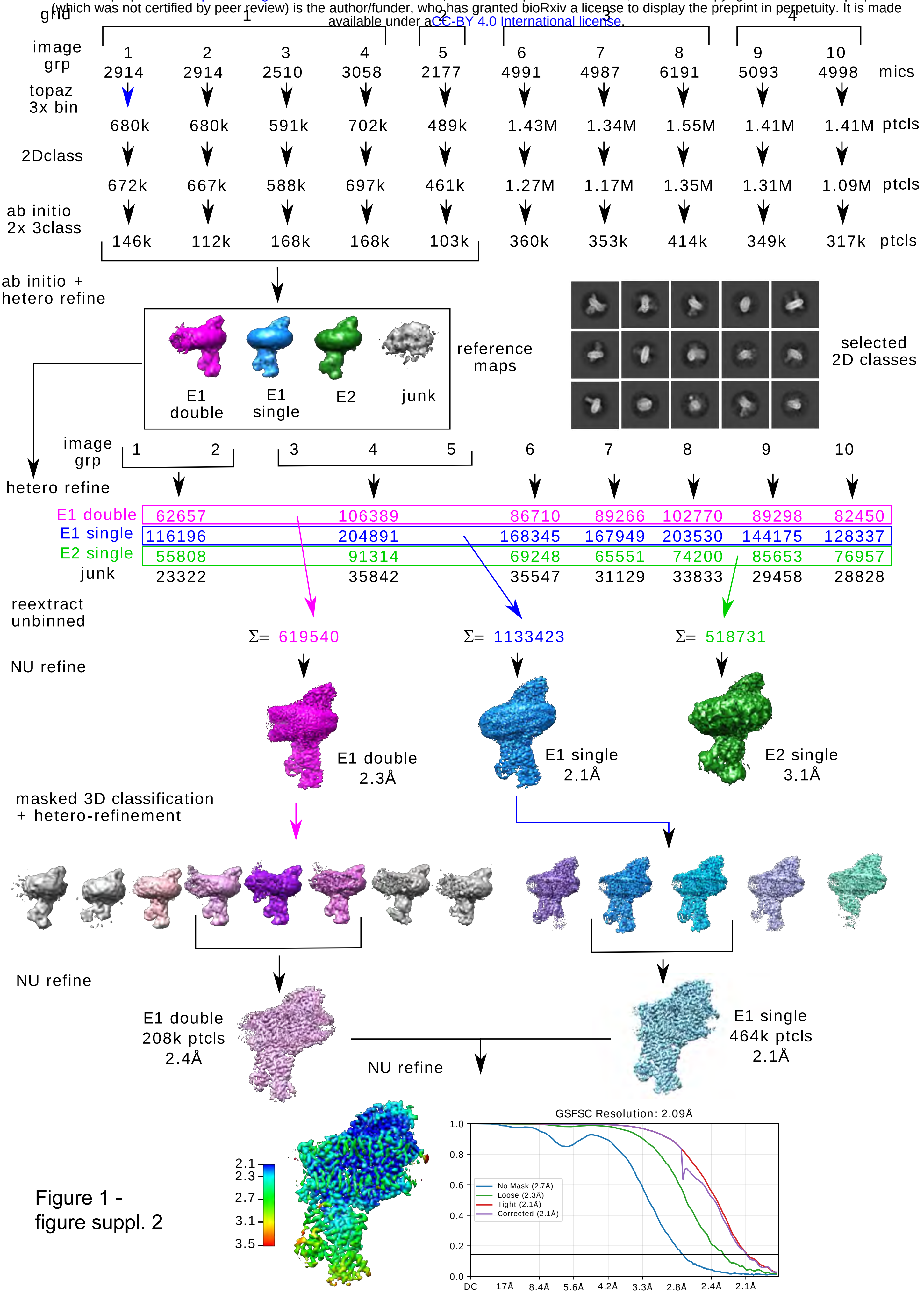


Figure 1 - figure suppl. 2

bioRxiv preprint doi: https://doi.org/10.1101/2025.05.05.652293; this version posted October 6, 2025. The copyright holder for this preprint (which was not certified by peer review) is the author/funder, who has granted bioRxiv a license to display the preprint in perpetuity. It is made available under aCC-BY 4.0 International license.

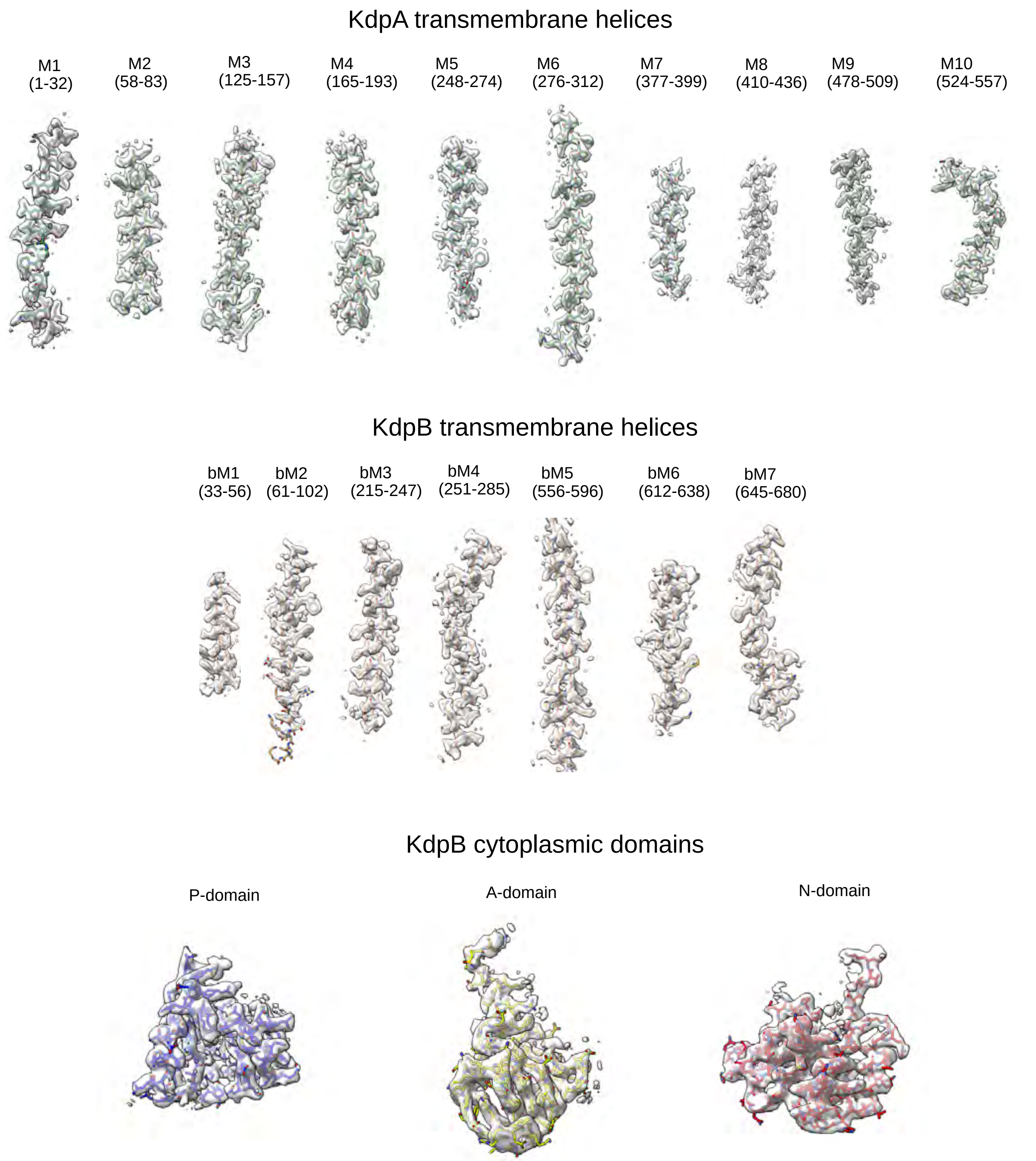


Figure 1 - figure supplement 3

bioRxiv preprint doi: https://doi.org/10.1101/2025.05.05.652293; this version posted October 6, 2025. The copyright holder for this preprint (which was not certified by peer review) is the author/funder, who has granted bioRxiv a license to display the preprint in perpetuity. It is made available under aCC-BY 4.0 International license.

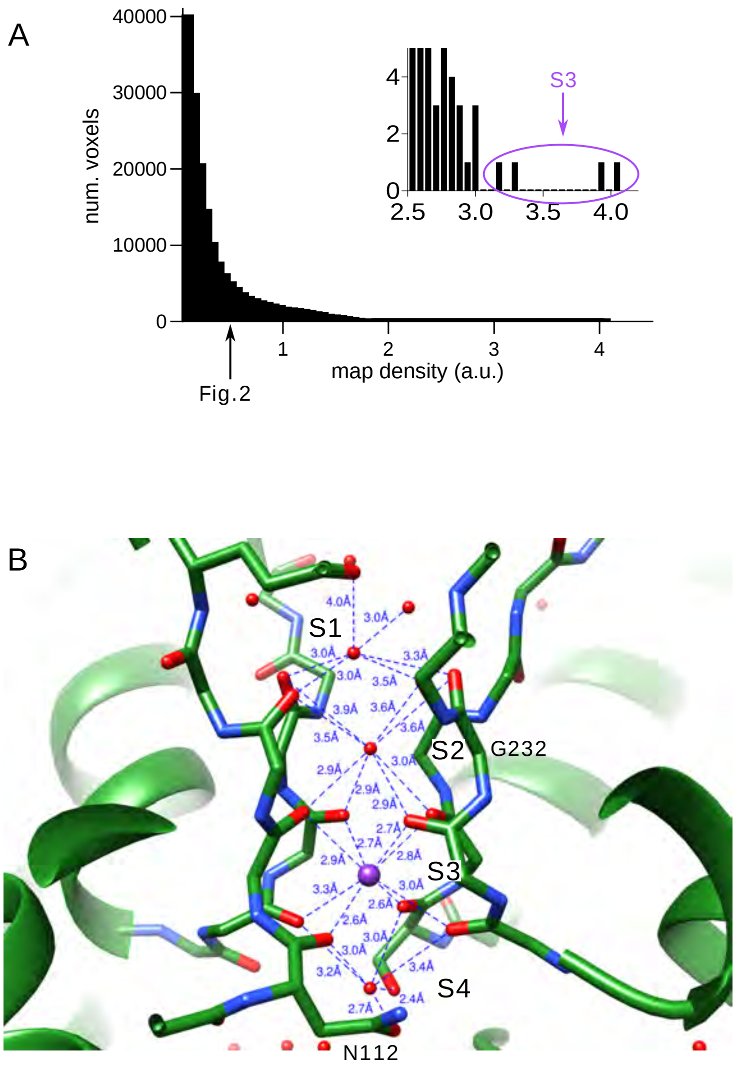


Figure 2 - figure supplement 1

bioRxiv preprint doi: https://doi.org/10.1101/2025.05.05.652293; this version posted October 6, 2025. The copyright holder for this preprint (which was not certified by peer review) is the author/funder, who has granted bioRxiv a license to display the preprint in perpetuity. It is made available under aCC-BY 4.0 International license.

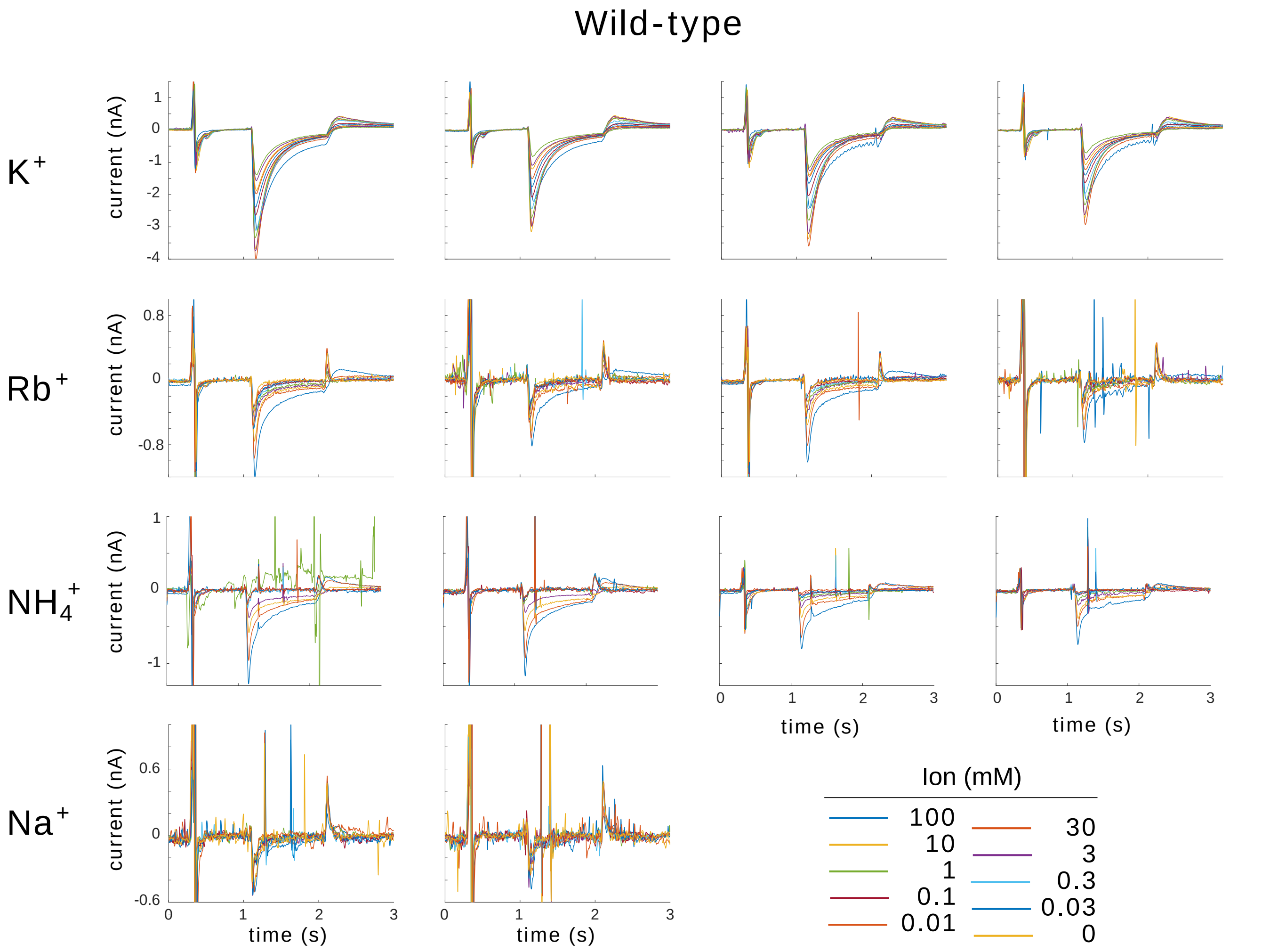


Figure 2 - figure supplement 2

bioRxiv preprint doi: https://doi.org/10.1101/2025.05.05.652293; this version posted October 6, 2025. The copyright holder for this preprint (which was not certified by peer review) is the author/funder, who has granted bioRxiv a license to display the preprint in perpetuity. It is made available under aCC-BY 4.0 International license.

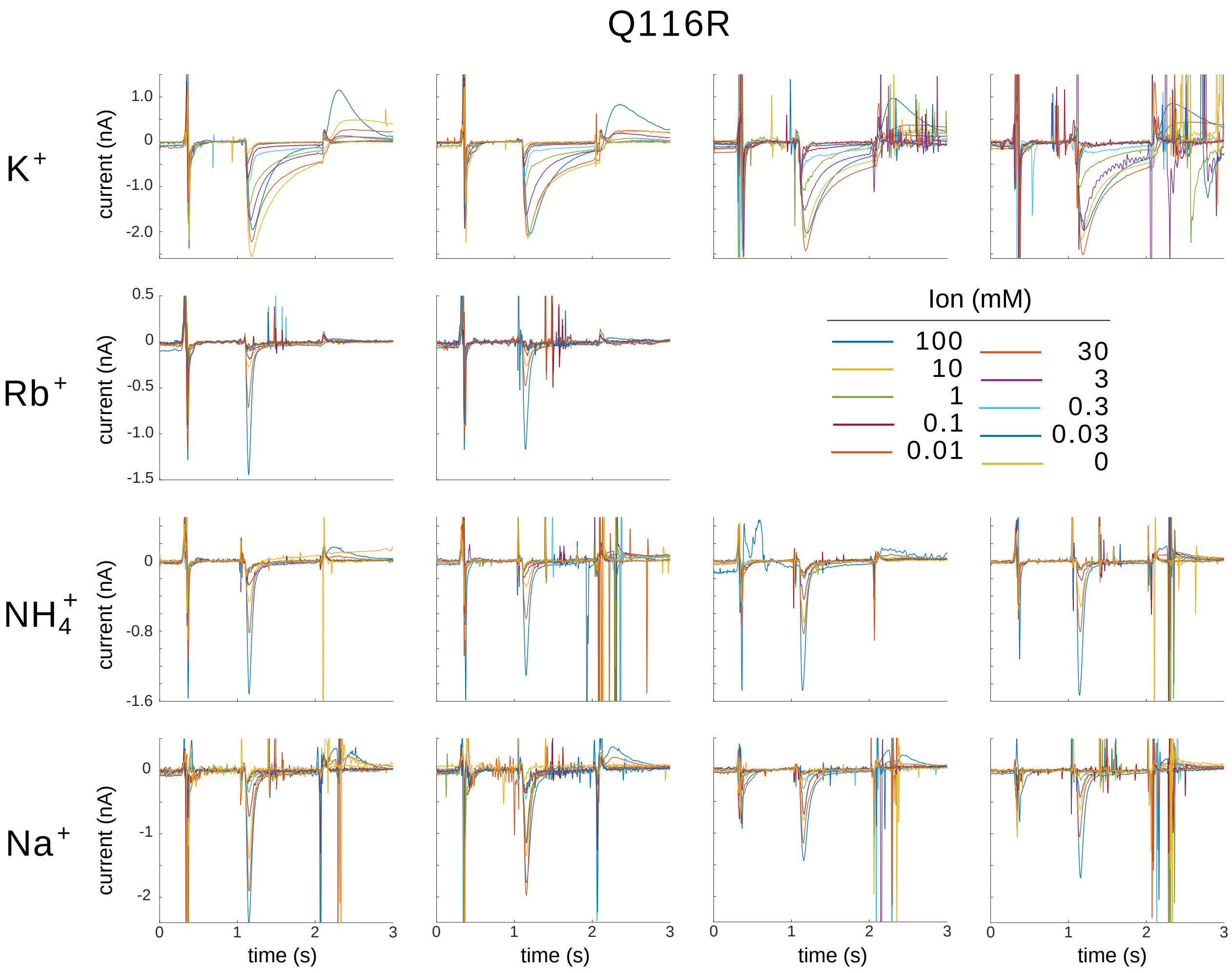


Figure 2 - figure supplement 3

bioRxiv preprint doi: https://doi.org/10.1101/2025.05.05.652293; this version posted October 6, 2025. The copyright holder for this preprint (which was not certified by peer review) is the author/funder, who has granted bioRxiv a license to display the preprint in perpetuity. It is made available under aCC-BY 4.0 International license.

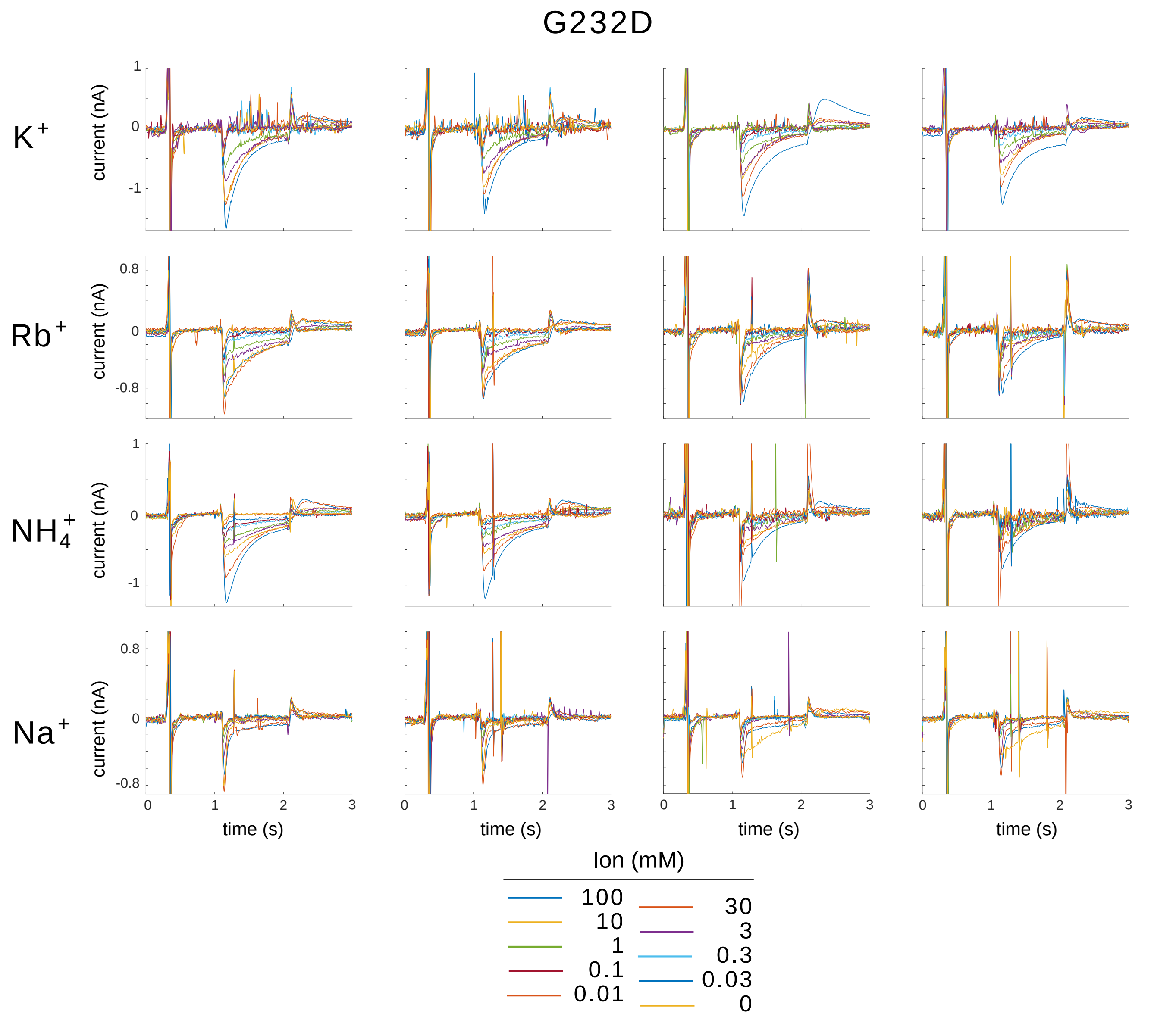


Figure 2 - figure supplement 4

bioRxiv preprint doi: https://doi.org/10.1101/2025.05.05.652293; this version posted October 6, 2025. The copyright holder for this preprint (which was not certified by peer review) is the author/funder, who has granted bioRxiv a license to display the preprint in perpetuity. It is made available under aCC-BY 4.0 International license.

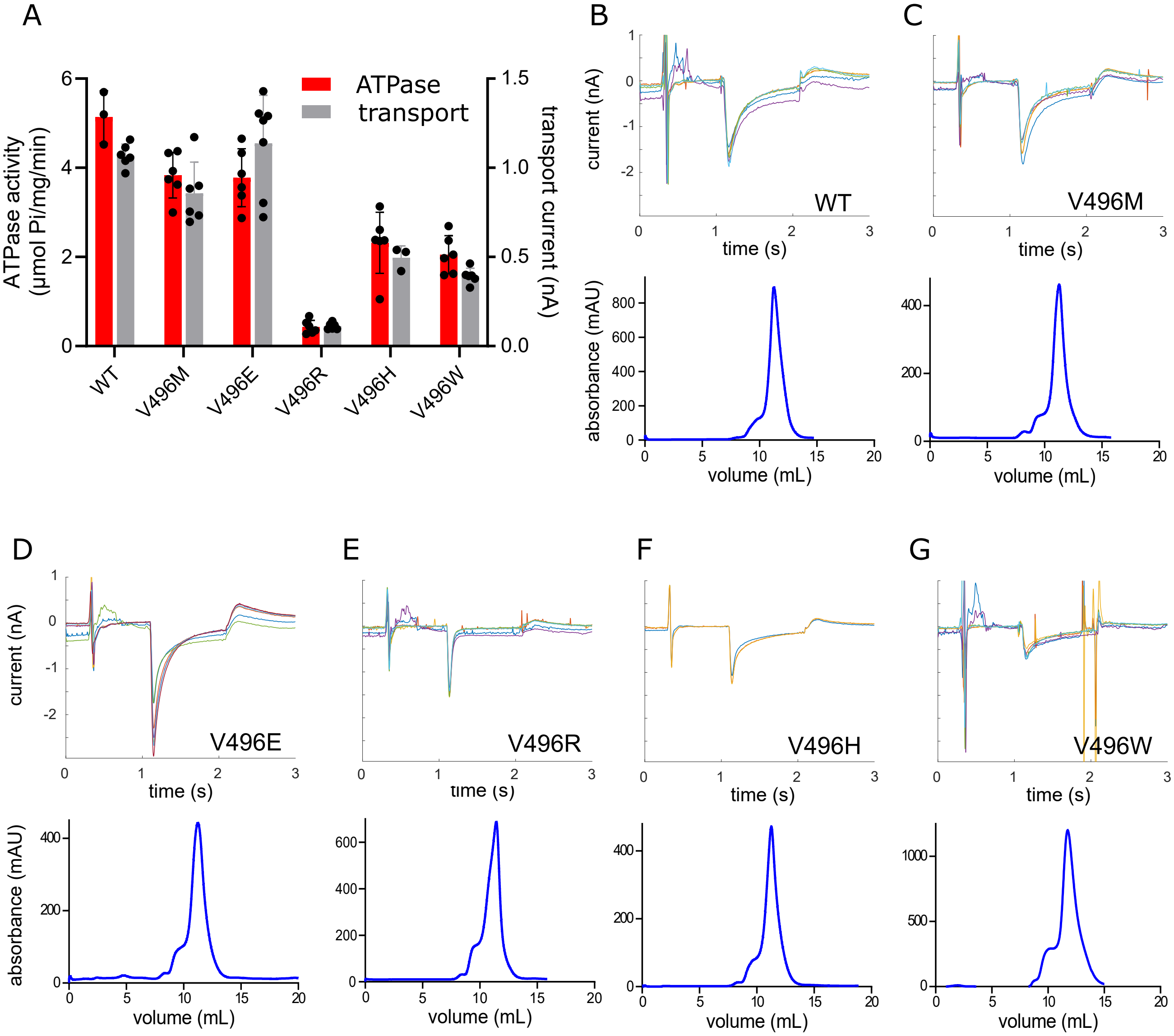


Figure 4 - figure supplement 1

bioRxiv preprint doi: https://doi.org/10.1101/2025.05.05.652293; this version posted October 6, 2025. The copyright holder for this preprint (which was not certified by peer review) is the author/funder, who has granted bioRxiv a license to display the preprint in perpetuity. It is made available under aCC-BY 4.0 International license.

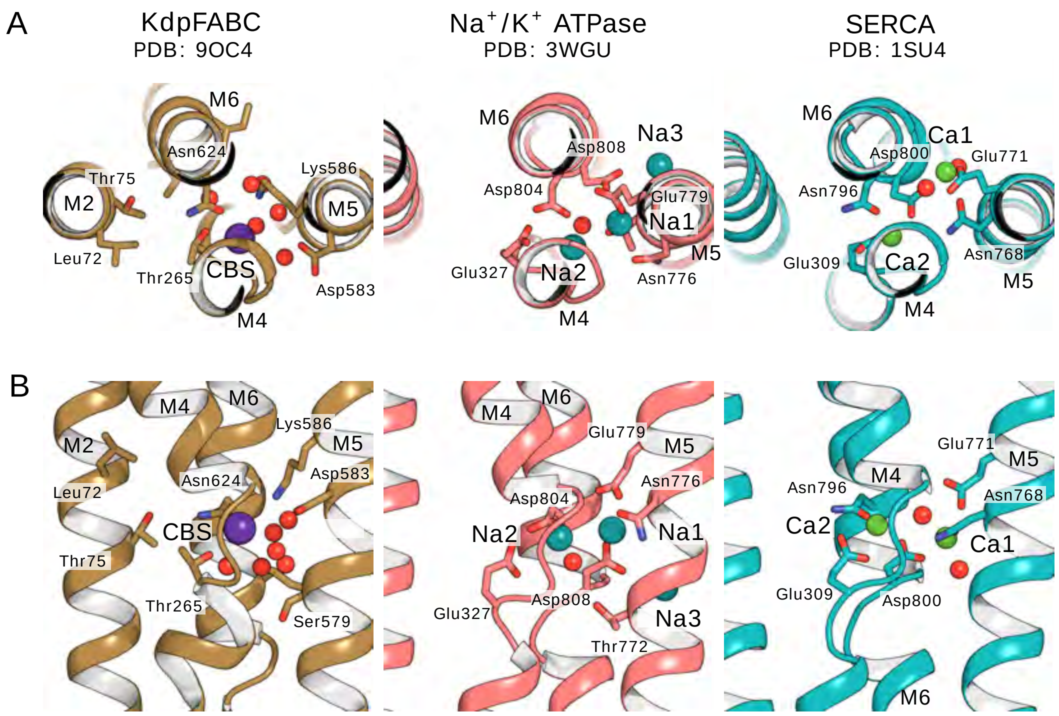


Figure 5 - figure supplement 1

bioRxiv preprint doi: https://doi.org/10.1101/2025.05.05.652293; this version posted October 6, 2025. The copyright holder for this preprint (which was not certified by peer review) is the author/funder, who has granted bioRxiv a license to display the preprint in perpetuity. It is made available under aCC-BY 4.0 International license.

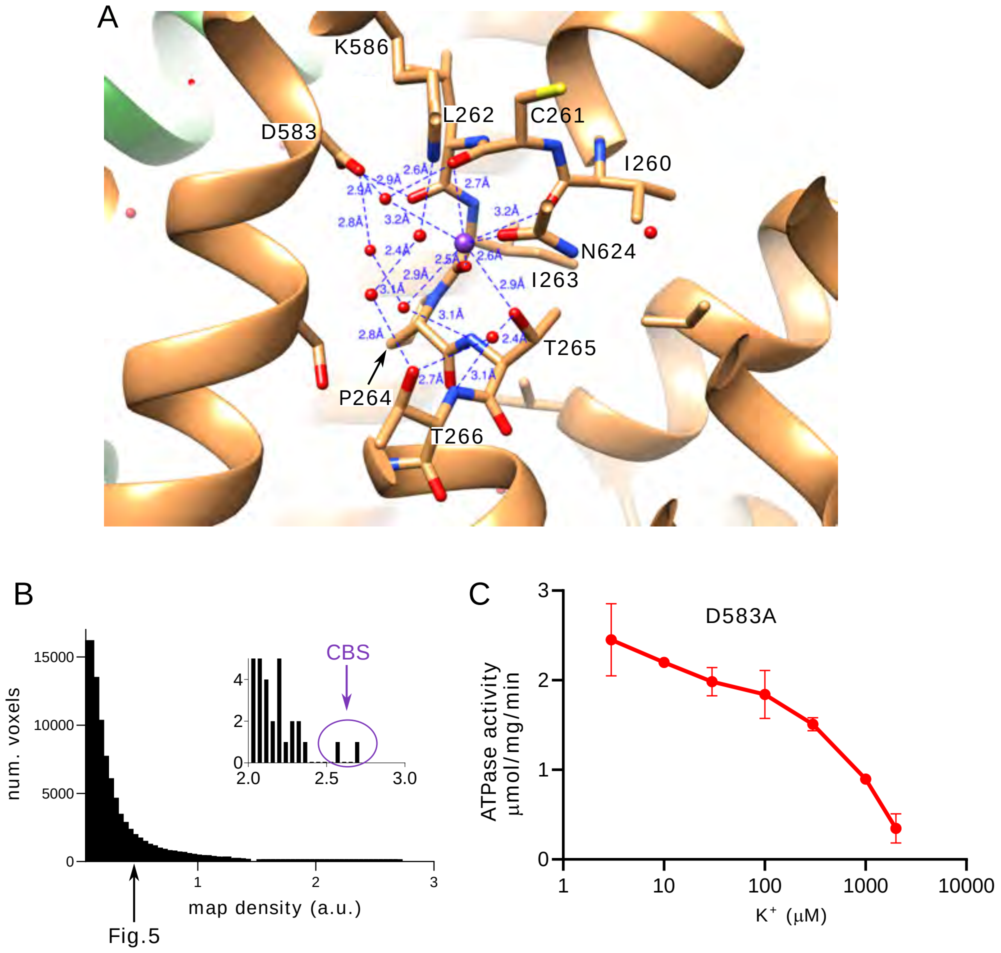


Figure 5 - figure supplement 2

bioRxiv preprint doi: https://doi.org/10.1101/2025.05.05.652293; this version posted October 6, 2025. The copyright holder for this preprint (which was not certified by peer review) is the author/funder, who has granted bioRxiv a license to display the preprint in perpetuity. It is made available under aCC-BY 4.0 International license.

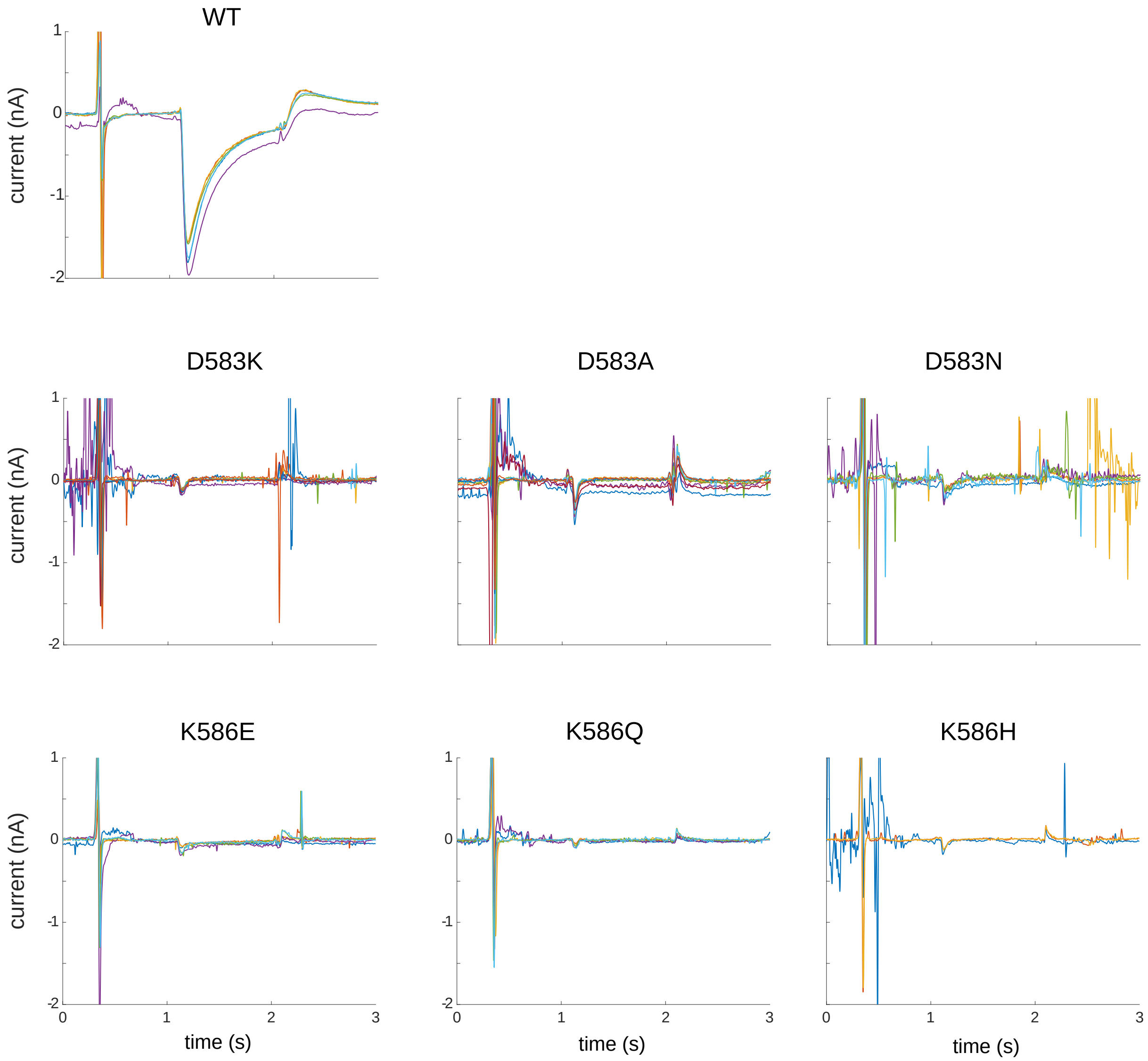


Figure 5 - figure supplement 3

bioRxiv preprint doi: https://doi.org/10.1101/2025.05.05.652293; this version posted October 6, 2025. The copyright holder for this preprint (which was not certified by peer review) is the author/funder, who has granted bioRxiv a license to display the preprint in perpetuity. It is made available under aCC-BY 4.0 International license.

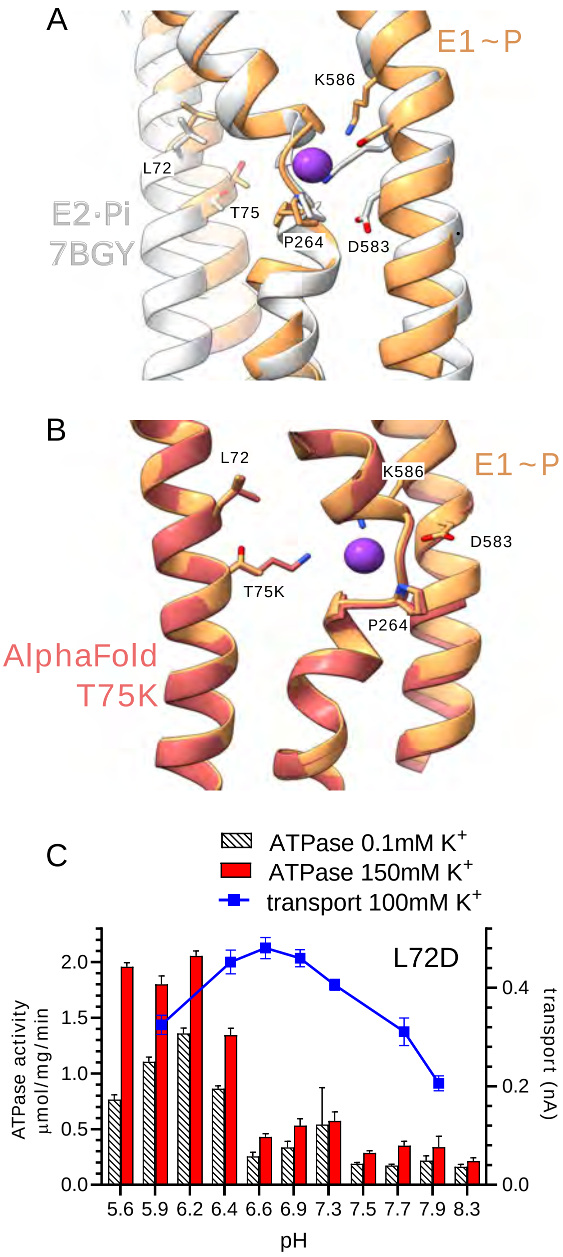


Figure 6 - figure supplement 1

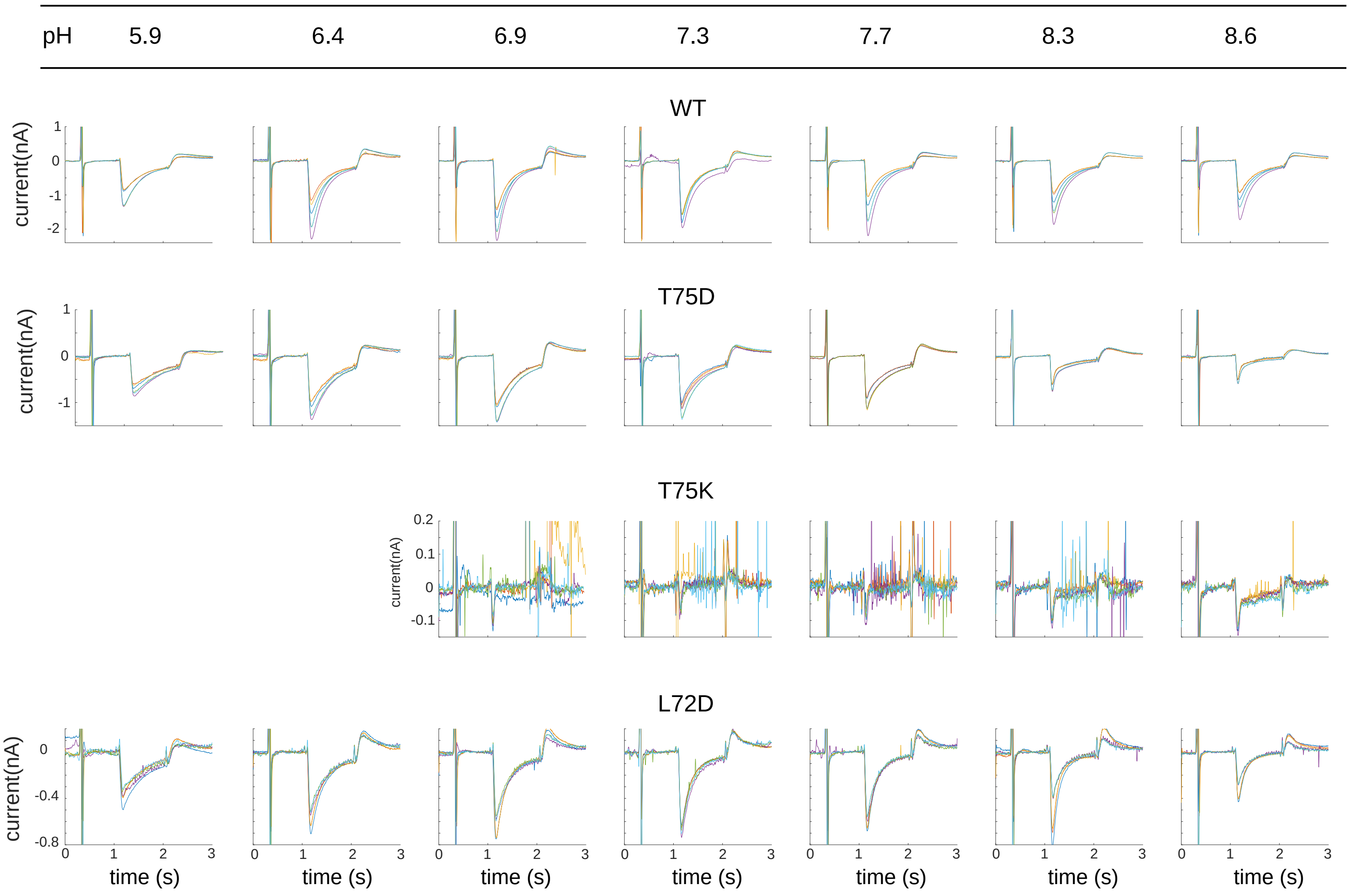


Figure 6 - figure supplement 2
