# Supplementary material for: Conduction pathway for potassium through the E. coli pump KdpFABC
Source: bioRxiv. 2025 Aug 22:2025.05.05.652293. Originally published 2025 May 6. Preprint. [Version 2] doi: 10.1101/2025.05.05.652293 (PMC12247778; doi:10.1101/2025.05.05.652293)
Supplement: Supplementary file 1 [file NIHPP2025.05.05.652293v2-supplement-1.pdf]

bioRxiv preprint doi: https://doi.org/10.1101/2025.05.05.652293; this version posted August 22, 2025. The copyright holder for this preprint (which was not certified by peer review) is the author/funder, who has granted bioRxiv a license to display the preprint in perpetuity. It is made available under aCC-BY 4.0 International license.

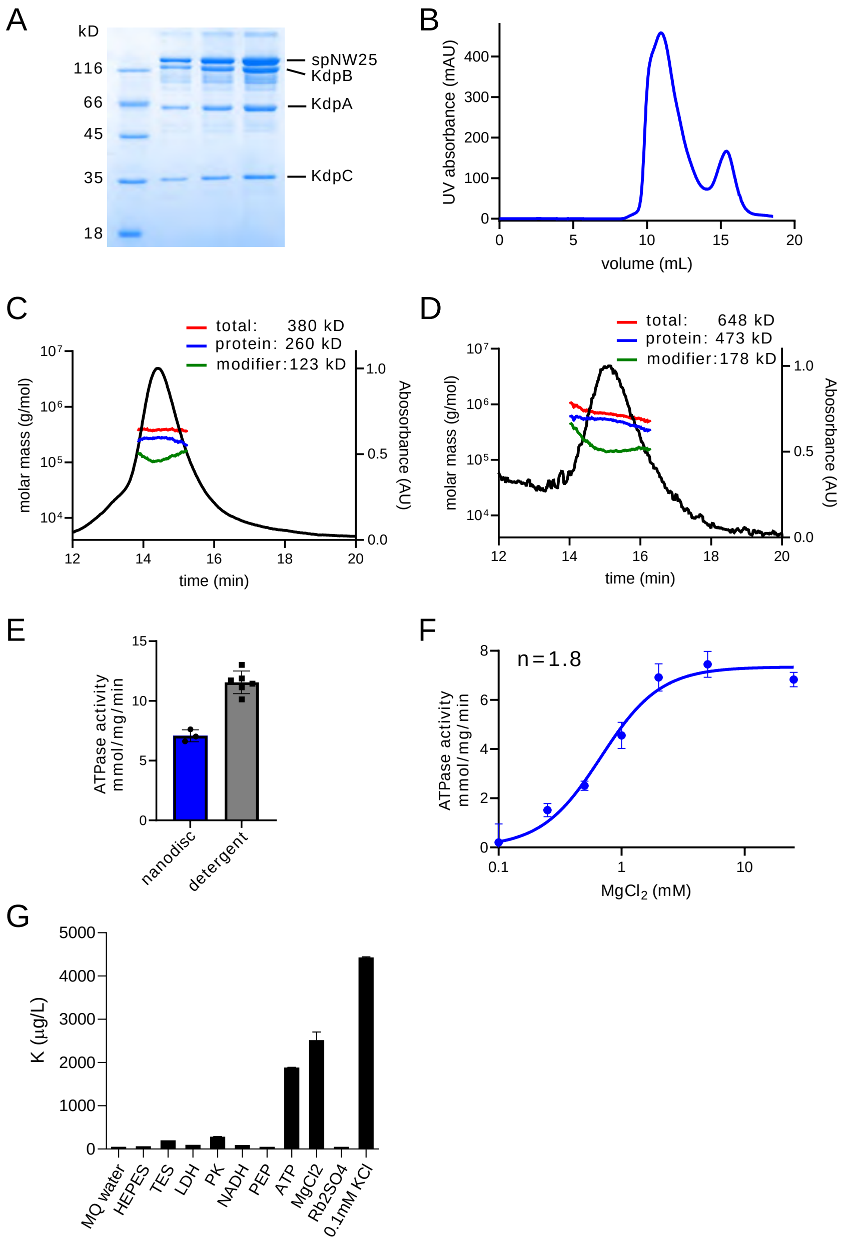

Figure 1 - figure supplement 1

bioRxiv preprint doi: https://doi.org/10.1101/2025.05.05.652293; this version posted August 22, 2025. The copyright holder for this preprint (which was not certified by peer review) is the author/funder, who has granted bioRxiv a license to display the preprint in perpetuity. It is made available under aCC-BY 4.0 International license.

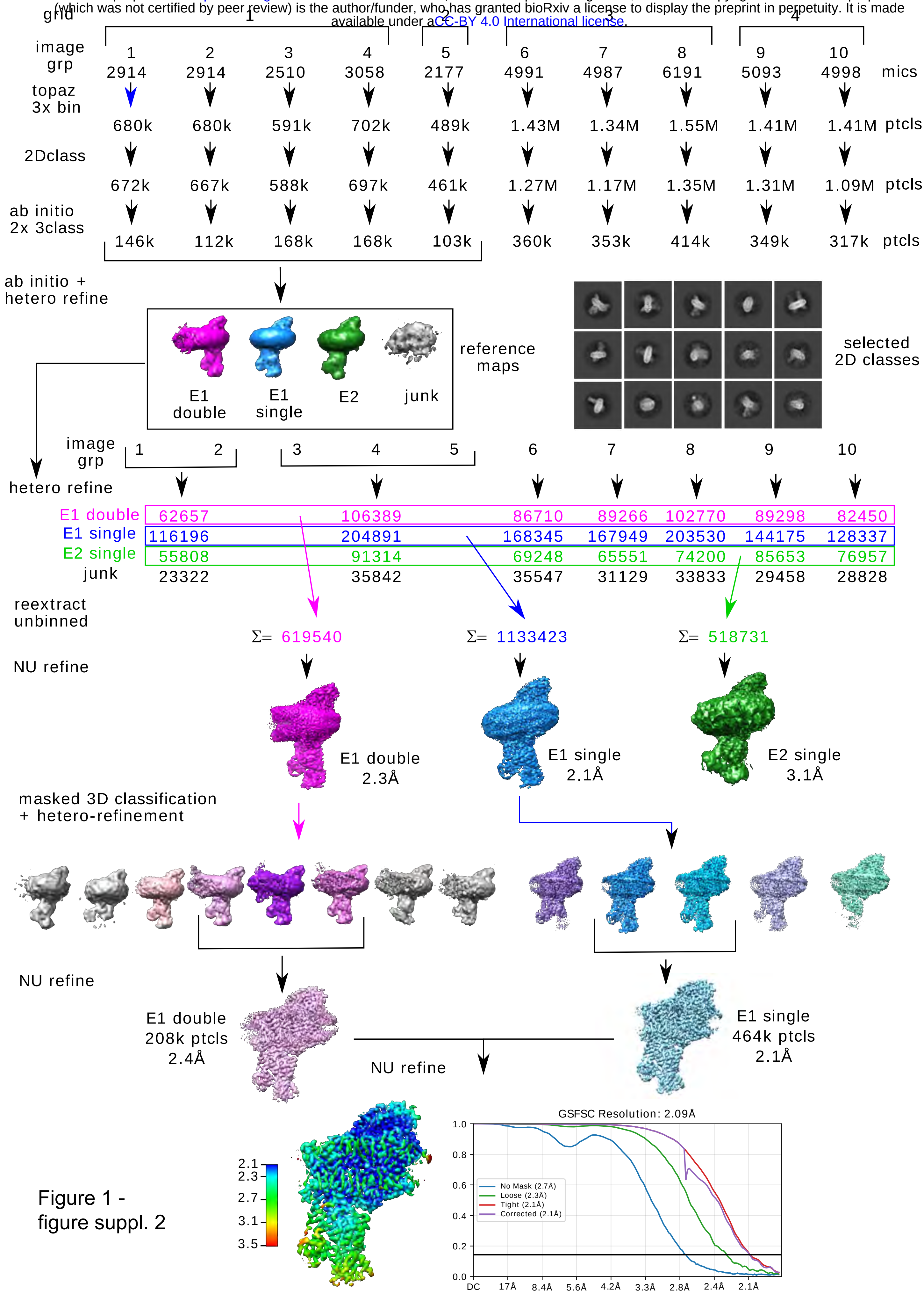

image grp	1	2	3	4	5	6	7	8	9	10
grid	1				2	3			4	
mics	2914	2914	2510	3058	2177	4991	4987	6191	5093	4998
ptcls (topaz 3x bin)	680k	680k	591k	702k	489k	1.43M	1.34M	1.55M	1.41M	1.41M
ptcls (2Dclass)	672k	667k	588k	697k	461k	1.27M	1.17M	1.35M	1.31M	1.09M
ptcls (ab initio 2x 3class)	146k	112k	168k	168k	103k	360k	353k	414k	349k	317k

hetero refine	1–2	3–5	6	7	8	9	10
E1 double	62657	106389	86710	89266	102770	89298	82450
E1 single	116196	204891	168345	167949	203530	144175	128337
E2 single	55808	91314	69248	65551	74200	85653	76957
junk	23322	35842	35547	31129	33833	29458	28828

Figure 1 - figure suppl. 2

bioRxiv preprint doi: https://doi.org/10.1101/2025.05.05.652293; this version posted August 22, 2025. The copyright holder for this preprint (which was not certified by peer review) is the author/funder, who has granted bioRxiv a license to display the preprint in perpetuity. It is made available under aCC-BY 4.0 International license.

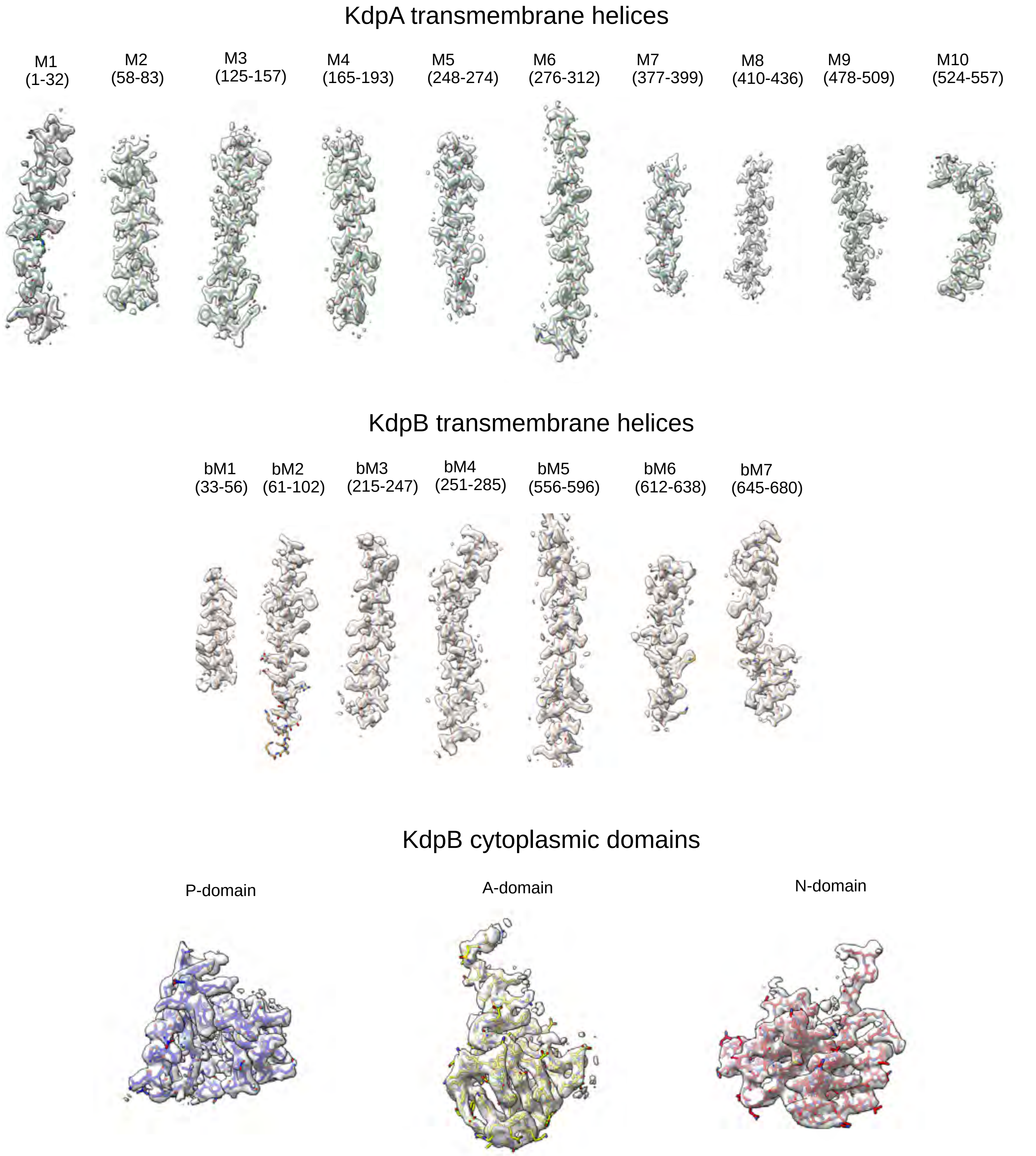

Figure 1 - figure supplement 3

bioRxiv preprint doi: https://doi.org/10.1101/2025.05.05.652293; this version posted August 22, 2025. The copyright holder for this preprint (which was not certified by peer review) is the author/funder, who has granted bioRxiv a license to display the preprint in perpetuity. It is made available under aCC-BY 4.0 International license.

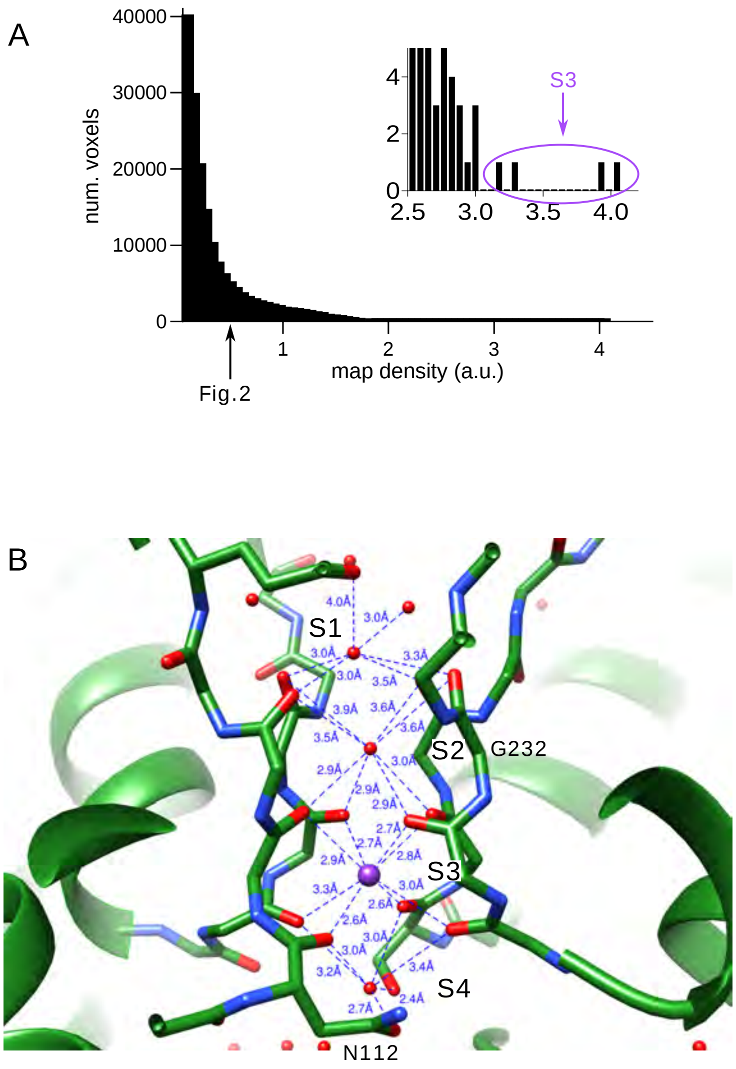

Figure 2 - figure supplement 1

bioRxiv preprint doi: https://doi.org/10.1101/2025.05.05.652293; this version posted August 22, 2025. The copyright holder for this preprint (which was not certified by peer review) is the author/funder, who has granted bioRxiv a license to display the preprint in perpetuity. It is made available under aCC-BY 4.0 International license.

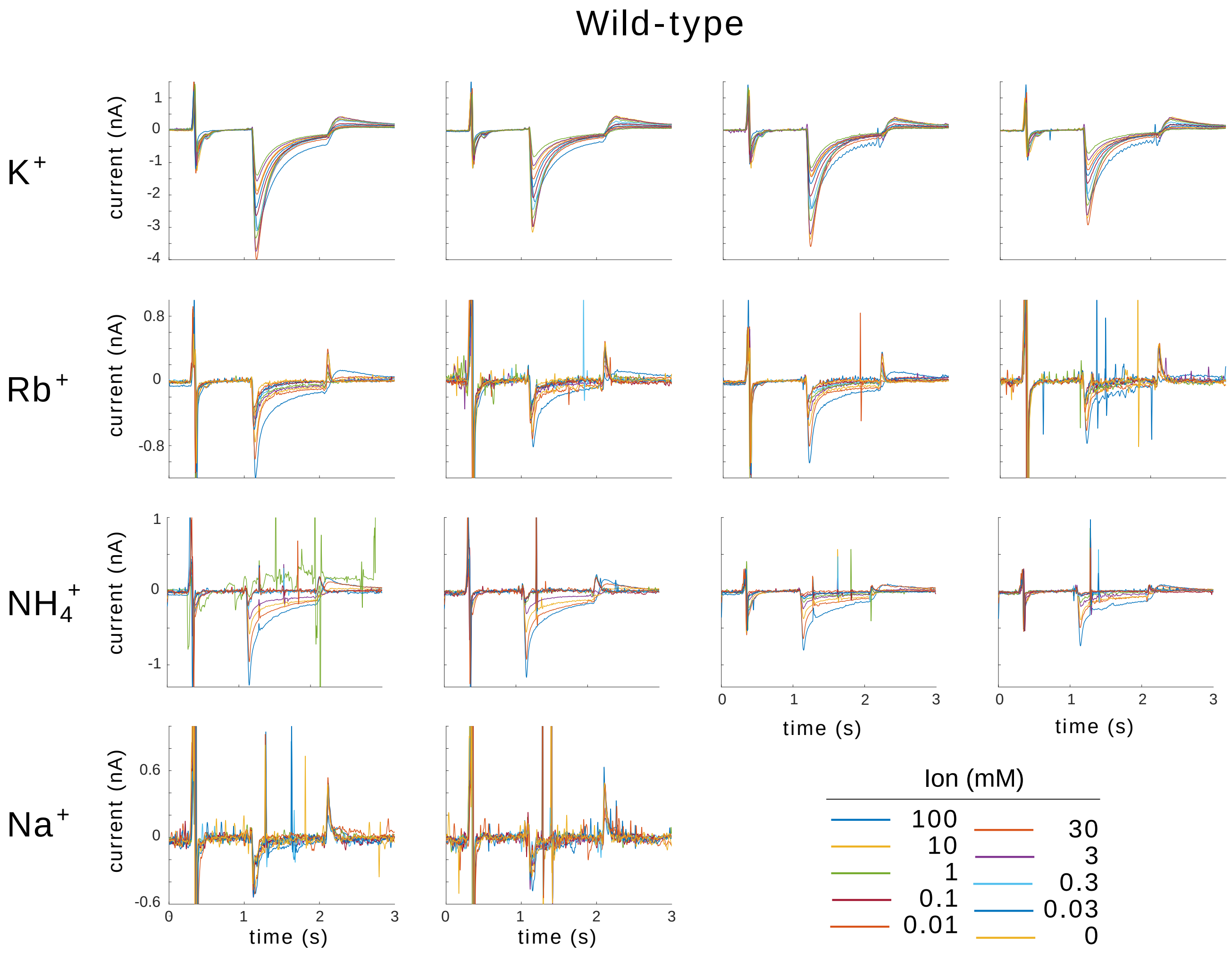

Figure 2 - figure supplement 2

bioRxiv preprint doi: https://doi.org/10.1101/2025.05.05.652293; this version posted August 22, 2025. The copyright holder for this preprint (which was not certified by peer review) is the author/funder, who has granted bioRxiv a license to display the preprint in perpetuity. It is made available under aCC-BY 4.0 International license.

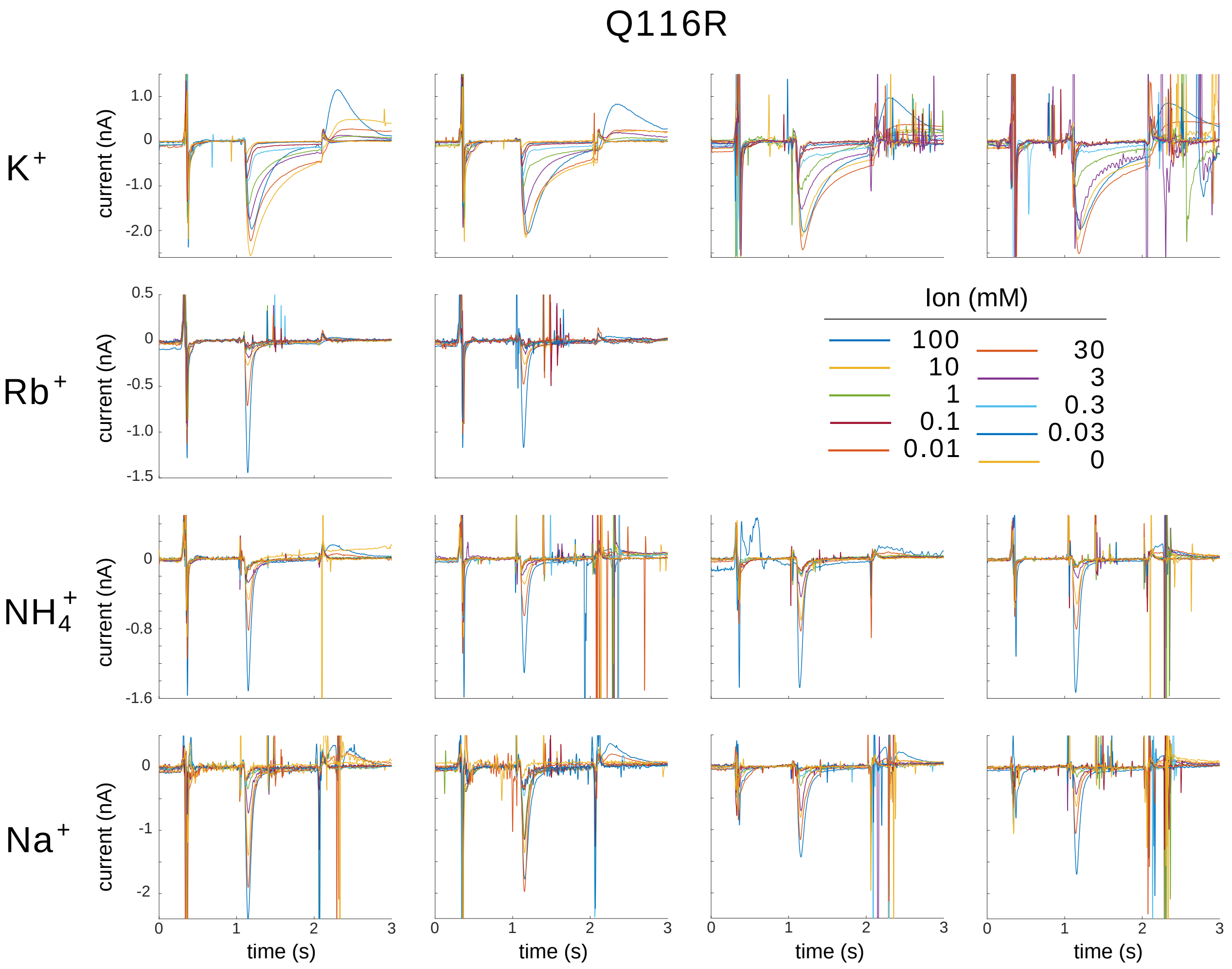

Figure 2 - figure supplement 3

bioRxiv preprint doi: https://doi.org/10.1101/2025.05.05.652293; this version posted August 22, 2025. The copyright holder for this preprint (which was not certified by peer review) is the author/funder, who has granted bioRxiv a license to display the preprint in perpetuity. It is made available under aCC-BY 4.0 International license.

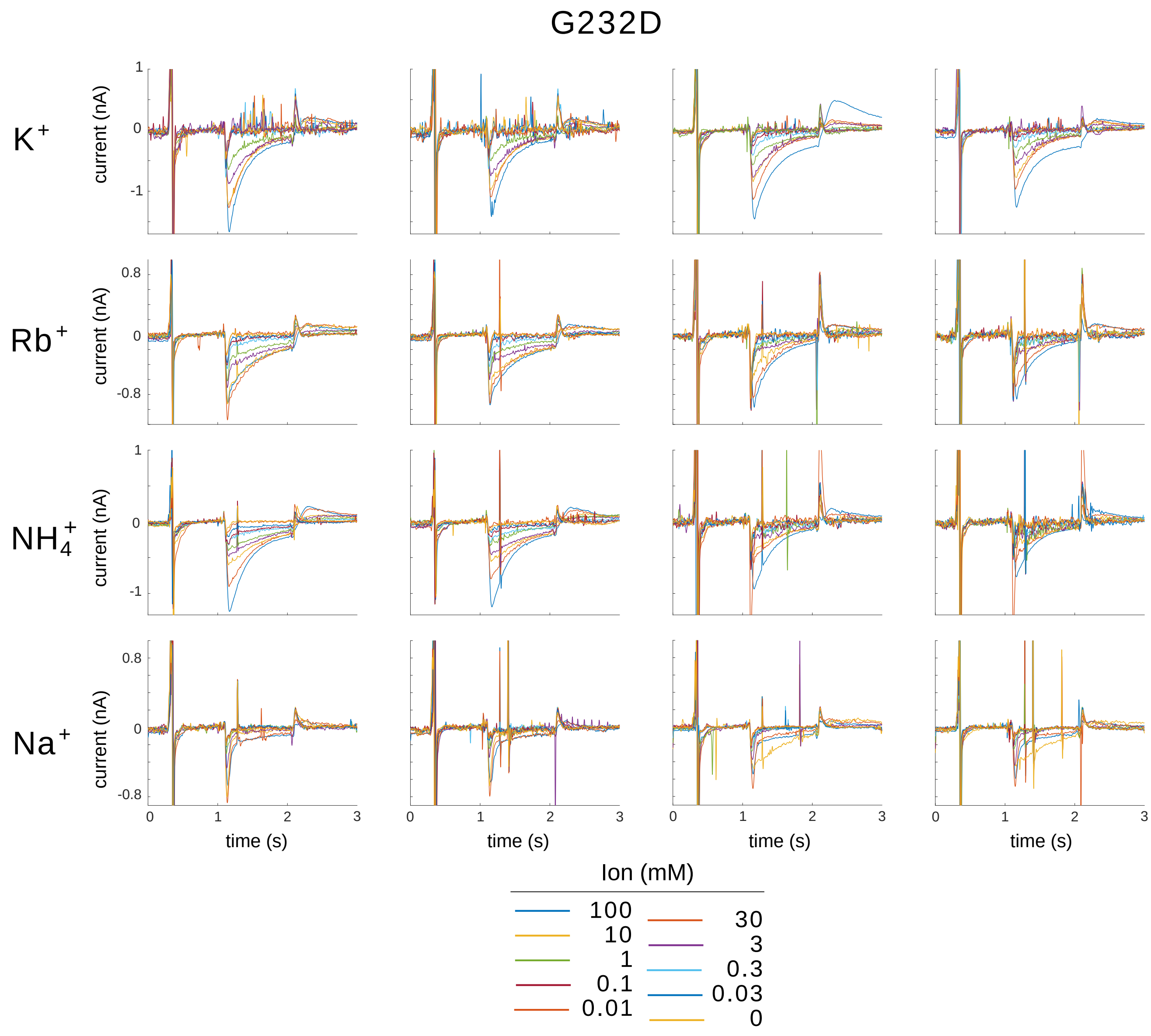

Figure 2 - figure supplement 4

bioRxiv preprint doi: https://doi.org/10.1101/2025.05.05.652293; this version posted August 22, 2025. The copyright holder for this preprint (which was not certified by peer review) is the author/funder, who has granted bioRxiv a license to display the preprint in perpetuity. It is made available under aCC-BY 4.0 International license.

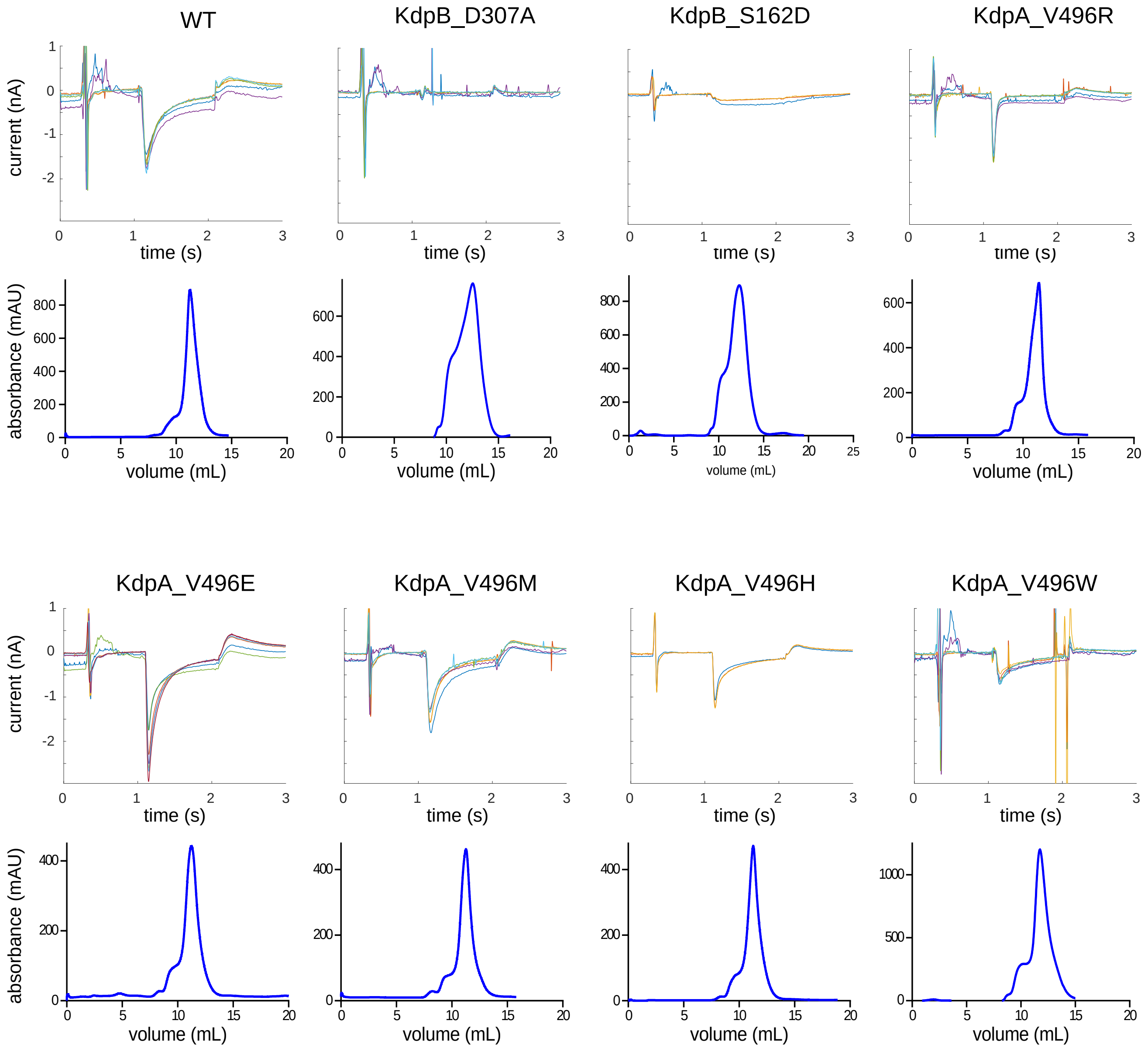

Figure 4 - figure supplement 1

bioRxiv preprint doi: https://doi.org/10.1101/2025.05.05.652293; this version posted August 22, 2025. The copyright holder for this preprint (which was not certified by peer review) is the author/funder, who has granted bioRxiv a license to display the preprint in perpetuity. It is made available under aCC-BY 4.0 International license.

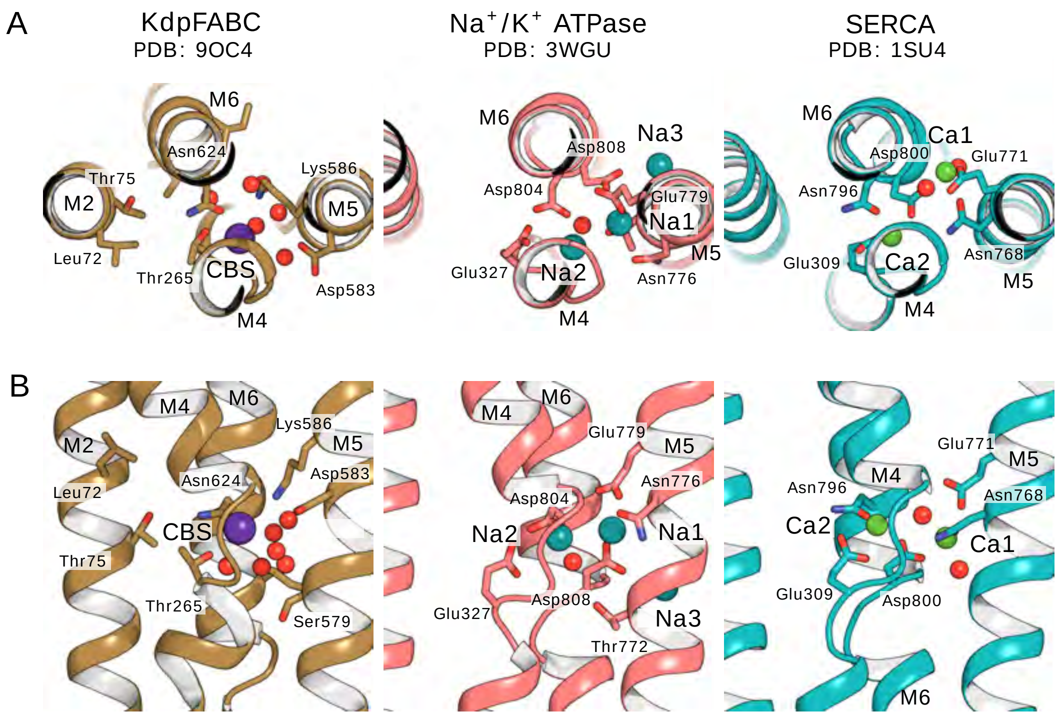

Figure 5 - figure supplement 1

bioRxiv preprint doi: https://doi.org/10.1101/2025.05.05.652293; this version posted August 22, 2025. The copyright holder for this preprint (which was not certified by peer review) is the author/funder, who has granted bioRxiv a license to display the preprint in perpetuity. It is made available under aCC-BY 4.0 International license.

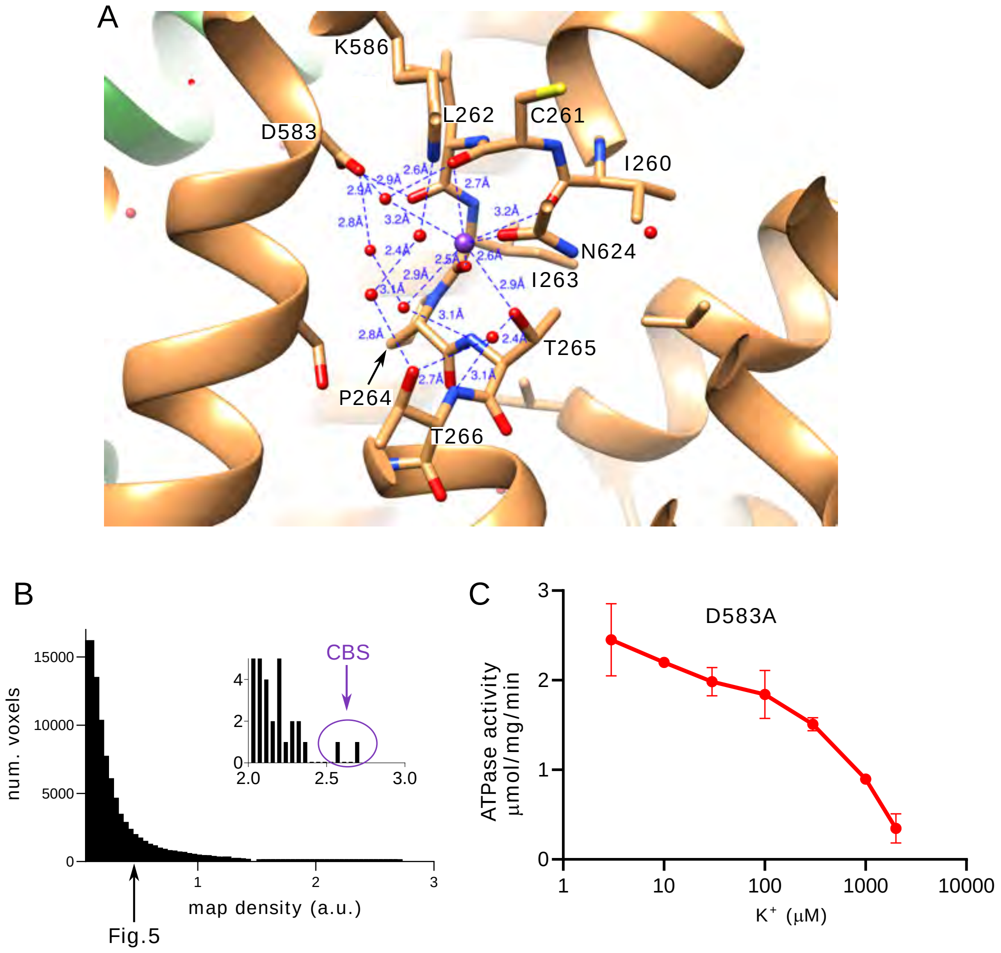

Figure 5 - figure supplement 2

bioRxiv preprint doi: https://doi.org/10.1101/2025.05.05.652293; this version posted August 22, 2025. The copyright holder for this preprint (which was not certified by peer review) is the author/funder, who has granted bioRxiv a license to display the preprint in perpetuity. It is made available under aCC-BY 4.0 International license.

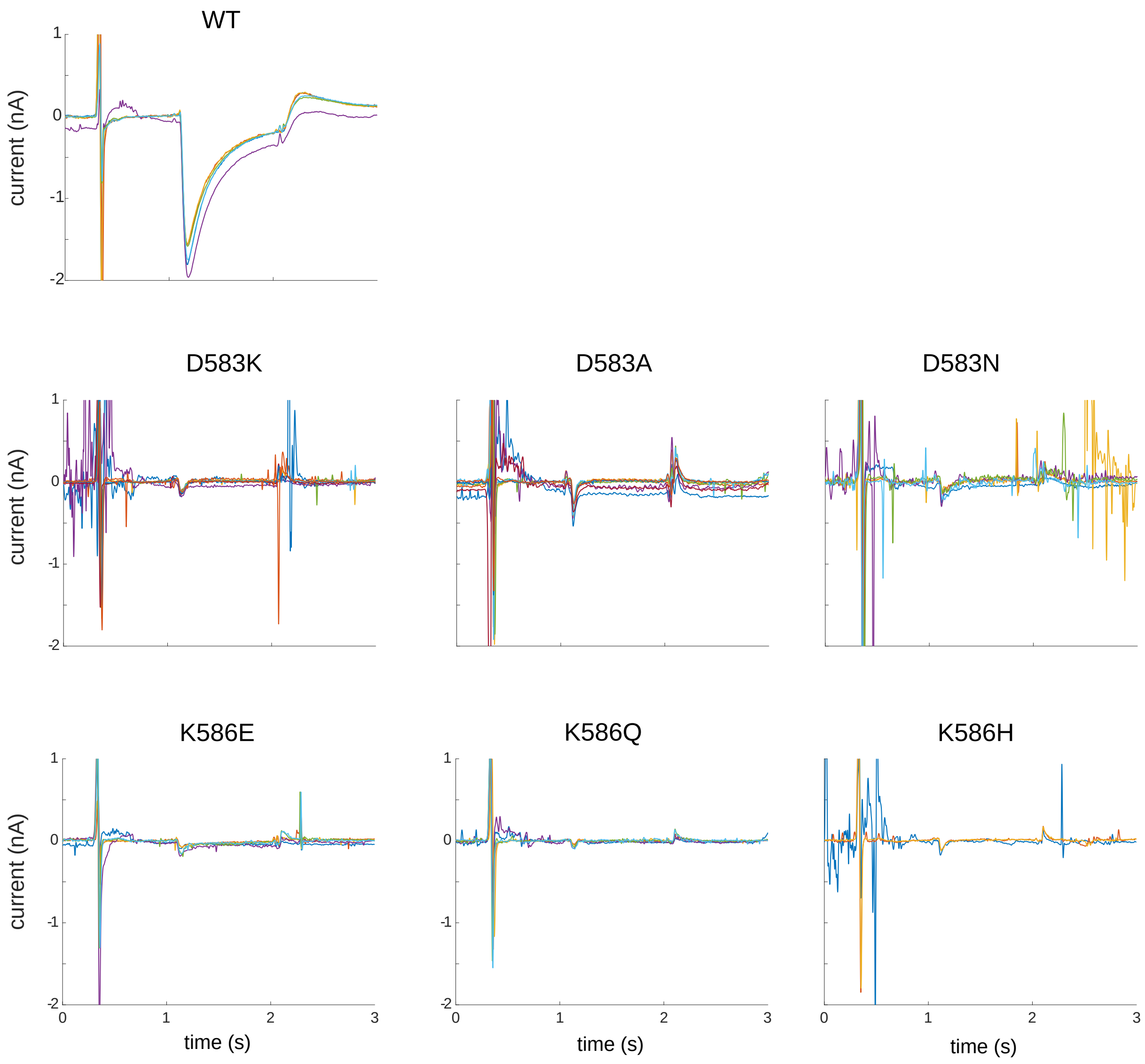

Figure 5 - figure supplement 3

bioRxiv preprint doi: https://doi.org/10.1101/2025.05.05.652293; this version posted August 22, 2025. The copyright holder for this preprint (which was not certified by peer review) is the author/funder, who has granted bioRxiv a license to display the preprint in perpetuity. It is made available under aCC-BY 4.0 International license.

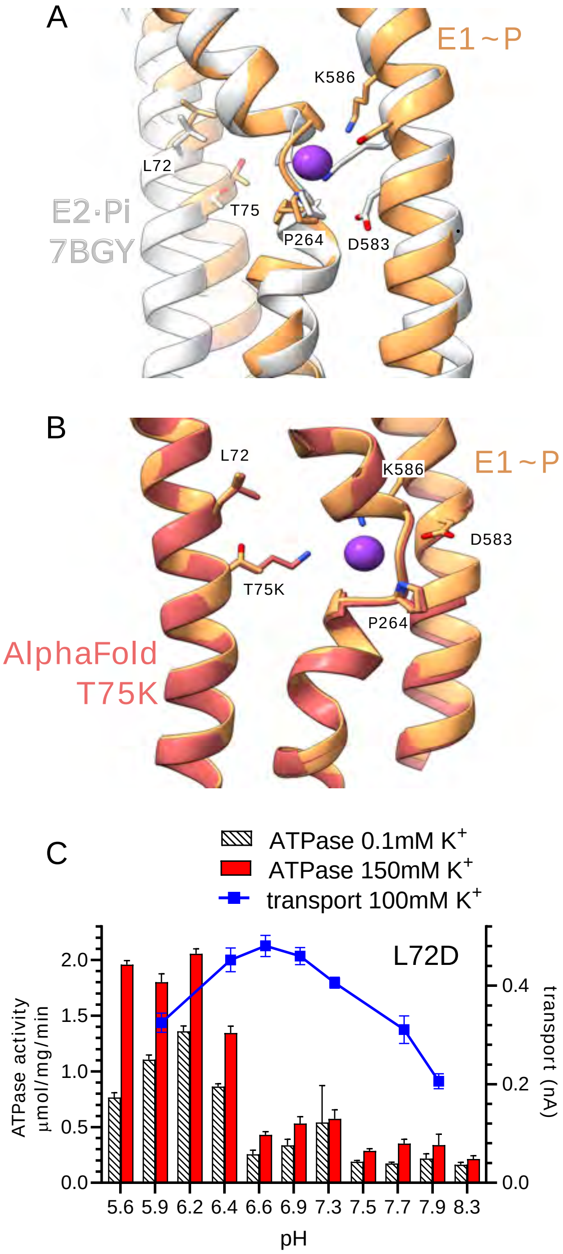

Figure 6 - figure supplement 1

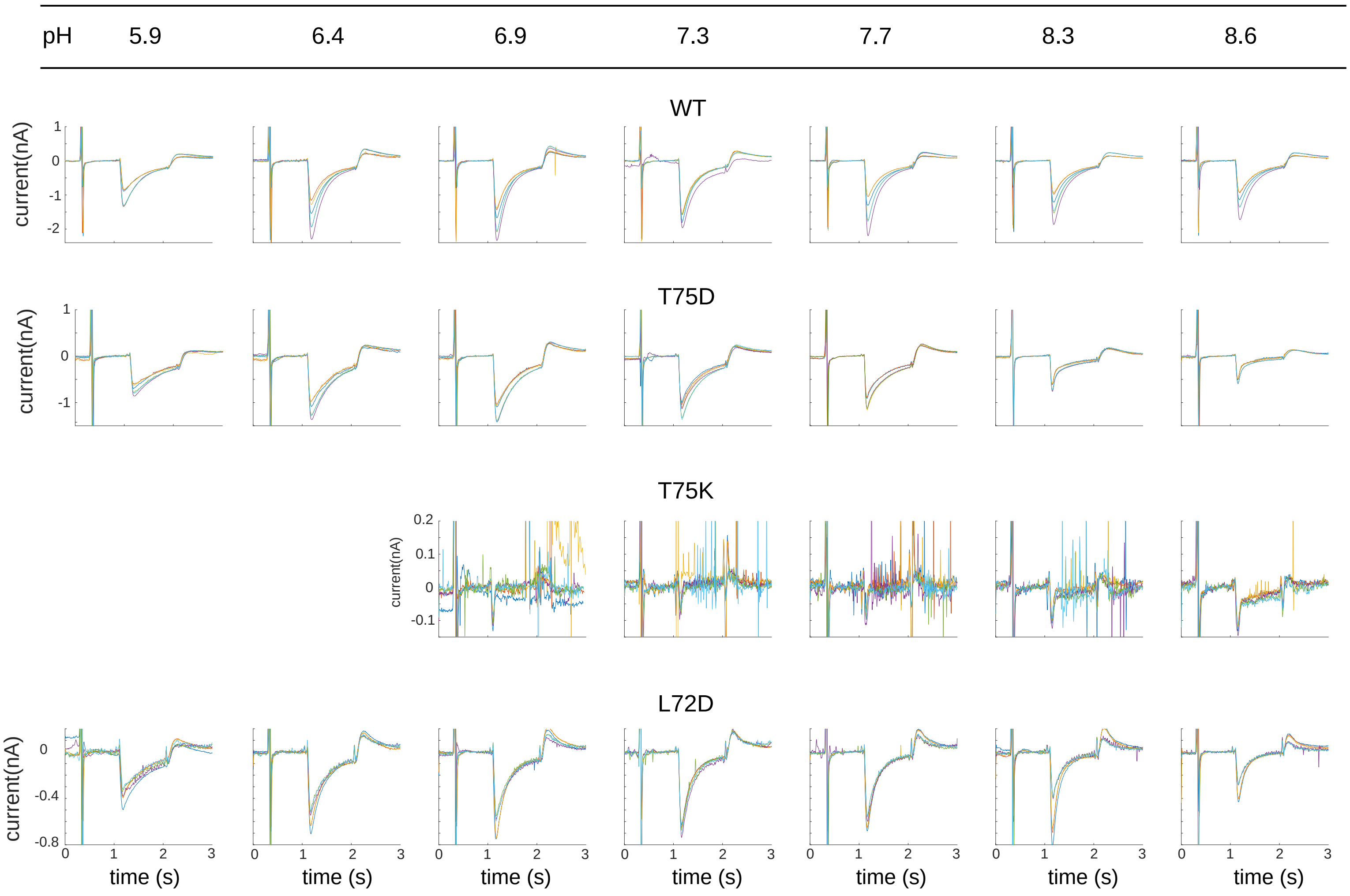

Figure 6 - figure supplement 2
